# Comprehensive Characterization of Androgen-Responsive lncRNAs Mediated Regulatory Network in Hormone-Related Cancers

**DOI:** 10.1155/2020/8884450

**Published:** 2020-09-26

**Authors:** Dan Wang, Mingyue Li, Jing Li, Xuechao Wan, Yan Huang, Chenji Wang, Pu Zhang, Yangguang Xu, Zhe Kong, Yali Lu, Xinmei Wang, Chuan Liu, Chaoneng Ji, Liang Li

**Affiliations:** ^1^State Key Laboratory of Genetic Engineering, Shanghai Engineering Research Center of Industrial Microorganisms, School of Life Science, Fudan University, Shanghai 200433, China; ^2^Department of Rehabilitation Medicine, The Third Affiliated Hospital, Sun Yat-Sen University, Guangzhou 510620, China; ^3^Department of Pathology, Zibo Central Hospital, Zibo 2550036, China; ^4^Department of Thyroid and Breast Surgery, Zibo Central Hospital, Zibo 255036, China

## Abstract

The AR signaling pathway plays an important role in initiation and progression of many hormone-related cancers including prostate, bladder, kidney, lung, and breast cancer. However, the potential roles of androgen-responsive long noncoding RNAs (lncRNAs) in hormone-related cancers remained unclear. In the present study, we identified 469 novel androgen-responsive lncRNAs using microarray data. After validating the accuracy of the array data, we constructed a transcriptional network which contained more than 30 transcriptional factors using ChIP-seq data to explore upstream regulators of androgen-responsive lncRNAs. Next, we conducted bioinformatics analysis to identify lncRNA-miRNA-mRNA regulatory network. To explore the potential roles of androgen-responsive lncRNAs in hormone-related cancers, we performed coexpression network and PPI network analyses using TCGA data. GO and KEGG analyses showed these lncRNAs were mainly involved in regulating signal transduction, transcription, development, cell adhesion, immune response, cell differentiation, and MAPK signaling pathway. We also highlight the prognostic value of HPN-AS1, TPTEP1, and LINC00623 in cancer outcomes. Our results suggest that androgen-responsive lncRNAs played important roles in regulating hormone-related cancer progression and could be novel molecular biomarkers.

## 1. Introduction

In the previous decade, one of the most interesting findings in the field of cancer biology was the novel annotation of noncoding RNA transcripts [1]. Long noncoding RNAs (lncRNAs), a kind of transcripts of more than 200 nucleotides without protein coding function, had been a rising star in cancer research. LncRNAs could act as oncogenes or tumor suppressors via regulating downstream targets at epigenetic [2], transcriptional [3], and posttranscriptional [4] levels. No more than 100 lncRNAs including PCAT-14 [5], NEAT1 [6], PCAT5 [7], and HOTAIR [8] were identified to play important roles in regulating tumor progression and dysregulated in different types of cancers (including prostate cancer, breast cancer, and kidney cancer). However, according to Sahu et al.'s report in 2015, more than 60000 lncRNAs located in the whole human genome [9]. Thus, the functional roles of most lncRNAs in cancers were still unknown.

Androgen/androgen receptor (AR) signaling has an important role in initiation and progression of many hormone-related cancers including prostate, bladder, kidney, lung, breast, and liver cancer [10]. Emerging evidences have demonstrated that androgen-responsive proteins and miRNAs regulated a series of cancer biological processes including cell cycle, apoptosis, and migration [11]. Our group and others have focused on exploring the molecular functions of androgen-responsive lncRNAs in PCa [12–14]. However, whether AR-regulated lncRNAs has differential roles in the individual cells within these tumors that contain a variety of cell types remains unclear. The understanding of the roles of AR downstream lncRNAs in many hormone-related tumors may eventually help us to develop better therapeutic approaches by targeting the AR.

In this study, we first identified novel androgen-responsive lncRNAs using microarray data. To explore upstream regulating factors, we constructed a transcriptional network using ChIP-seq data. Next, we conducted bioinformatics analysis to identify lncRNA-miRNA-mRNA regulatory network in hormone-related cancers. To explore the potential roles of androgen-responsive lncRNAs in hormone-related cancers, we performed coexpression network analysis and GO and KEGG analyses using TCGA data. Our results suggest that androgen-responsive lncRNAs played important roles in regulating hormone-related cancer progression and could act as novel molecular biomarkers.

## 2. Material and Methods

### 2.1. Cell Culture and Androgen Treatment

LNCaP cells were purchased from the American Type Culture Collection (Manassas, USA) which was confirmed by short tandem repeat (STR) analysis. All experiments were carried out by each cell line at passages below 30. LNCaP cells were maintained in RPMI 1640 medium (Corning, USA) supplemented with 10% FBS (Hyclone, USA) and cultured at 37°C in 5% CO_2_. Androgen treatment assay was performed as described previously [15].

### 2.2. Microarray and Expression Data Sets

Total RNA of samples were isolated by using TRIzol (Invitrogen) and the RNeasy mini kit (QIAGEN). Total RNA was quantified by the NanoDrop ND-2000 (Thermo Scientific), and the RNA integrity was assessed using Agilent Bioanalyzer 2100 (Agilent Technologies).

Total RNA from LNCaP cells, treated with 10 nM DHT or ethanol for 8 hours, were hybridized to SBC-ceRNA (4^∗^180 K). The sample labeling, microarray hybridization, and washing were performed based on the manufacturer's standard protocols. The raw microarray data are listed in supplementary table 1. To begin with, the raw data was normalized with the quantile algorithm. The probes that have at least 1 condition out of 2 conditions and have flags in “P” were chosen for further data analysis. Differentially expressed lncRNAs were then identified through fold change. The threshold set for up- and downregulated genes was a fold change ≥ 2.0.

### 2.3. Construction of Androgen-Responsive ceRNA Networks in PCa

**Figure 1:**
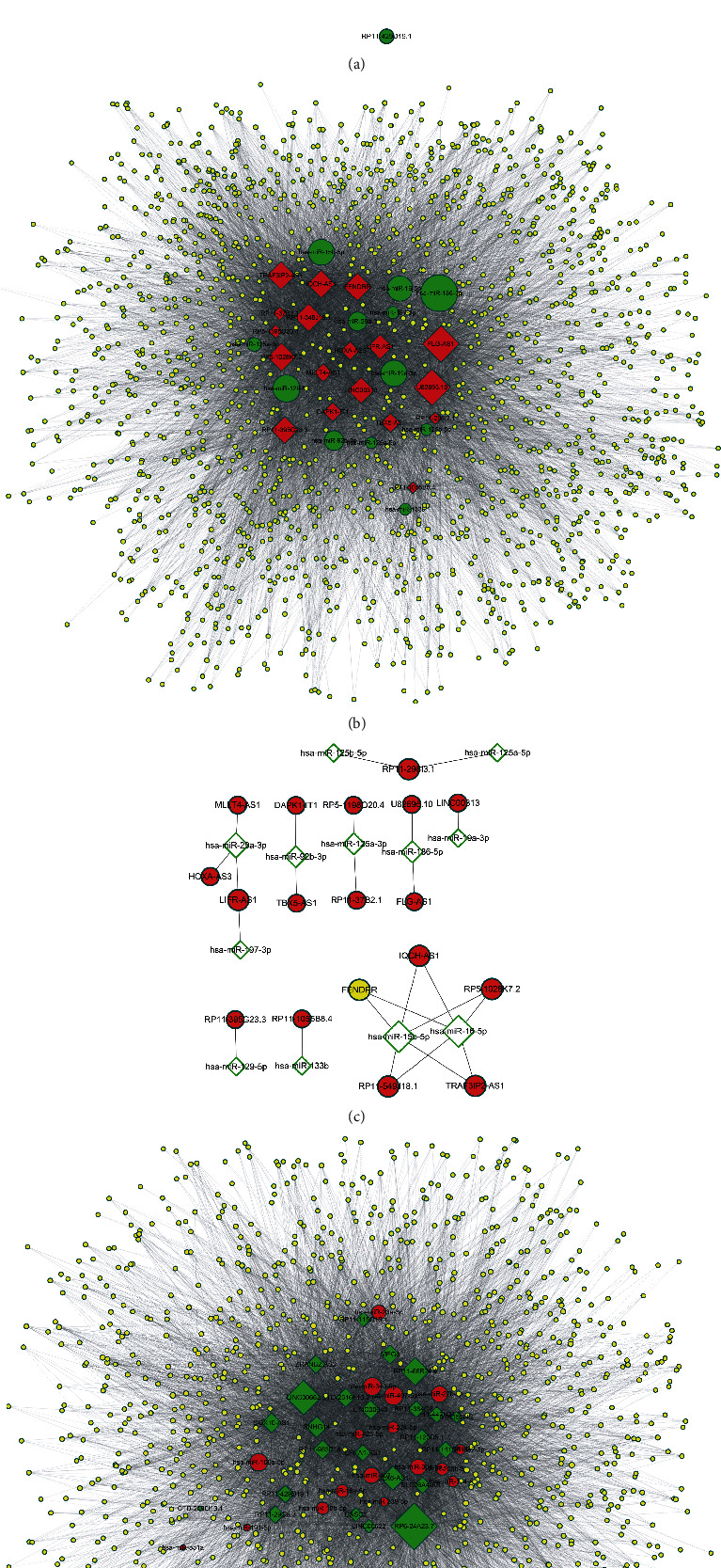
The predicted interactions between differentially expressed lncRNAs and their target miRNAs. We constructed androgen-induced lncRNAs-miRNA (a) or androgen-induced lncRNAs-miRNA-mRNA networks (b). (c, d) The predicted androgen-reduced lncRNAs-miRNA (c) or androgen-reduced lncRNAs-miRNA-mRNA (d) networks were constructed based on bioinformatics analysis.

We predicted the interactions between differentially expressed lncRNAs and their target miRNAs theoretically by using miRcode. Finally, TargetScan and StarBase databases were both used to identify miRNAs which suppress mRNAs. The networks were drawn using Cytoscape 3.0 (Figures 1(a)–1(d)).

### 2.4. Real-Time Reverse Transcription PCR (qRT-PCR) Analysis

qRT-PCR for lncRNAs and mRNAs was performed as described previously [16]. The Ct values were normalized using *β*-actin as internal control to estimate the different expression of genes. Relative mRNA expression was calculated using the 2^-*ΔΔ*Ct^ method. Each sample was run in triplicate to ensure quantitative accuracy.

### 2.5. Hierarchical Clustering Analysis

To generate an overview of lncRNA/mRNA expression profiles between the two groups, the hierarchical clustering analysis was performed based on the expression value of all targets and the most significant differentially expressed lncRNA/mRNAs using Cluster and TreeView program.

### 2.6. Gene Ontology (GO) and Pathway Analysis

We conducted Gene Ontology (GO) analysis to construct meaningful annotation of genes by using MAS system provided by CapitalBio company (Molecule Annotation System, http://bioinfo.capitalbio.com/mas3/). The ontology has covered domains of biological processes, cellular components, and molecular functions. The enriched GO terms were presented by enrichment scores. KEGG pathway analysis was carried out to determine the involvement of differentially expressed mRNAs in different biological pathways. *P* < 0.05 was used as the criterion for statistical significance.

### 2.7. Statistical Analysis

The numerical data was presented as mean ± standard deviation (SD) of at least three determinations. Statistical comparisons between groups of normalized data were performed using *T*-test or Mann–Whitney *U*-test according to the test condition. A *P* < 0.05 was considered statistical significance with a 95% confidence level.

## 3. Result

### 3.1. Identification of Differentially Expressed Androgen-Responsive lncRNAs in Prostate Cancer Patients

**Figure 2:**
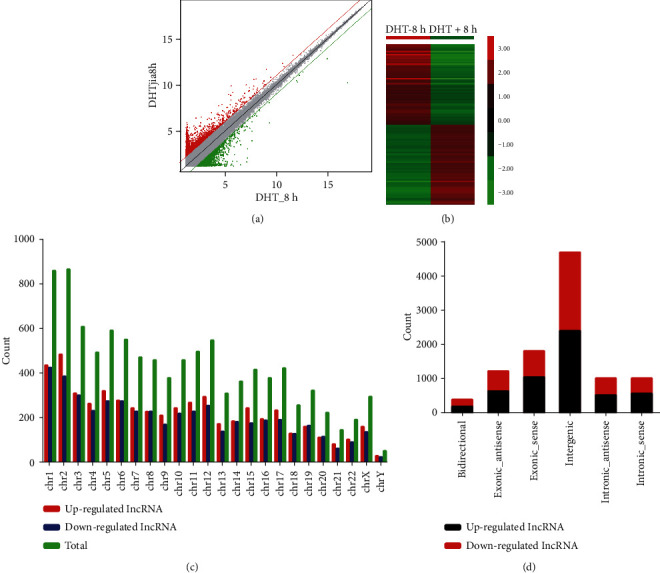
The microarray assay for androgen-responsive lncRNAs in LNCaP cells after 8 h treatment of DHT. (a) The volcano plot showing differentially expressed lncRNAs. (b) The heat map showing differentially expressed lncRNAs. (c) The statistics of androgen-responsive lncRNAs arranged by chromosome. (d) The classification of differentially expressed lncRNAs.

The workflow of this study is shown in supplementary description. High-throughput microarray assay was performed to identify androgen-responsive lncRNAs in LNCaP cells after 8 h treatment of DHT. The volcano plot filtering identified changed lncRNAs with statistical significance between two groups (Figure 2(a)). And the differentially expressed lncRNAs were displayed through fold change filtering (Figure 2(a)). Hierarchical clustering showed androgen-induced and reduced lncRNAs in PCa (Figure 2(b)).

As a result, 469 lncRNAs were detected to be differentially regulated by fold change ≥ 2.0, among which 285 lncRNAs were upregulated while 184 lncRNAs were downregulated. Among these lncRNAs, upregulated lncRNAs are more common than downregulated lncRNAs. The distribution of the lncRNAs on the human chromosomes is depicted, and we did not observe specific chromosomal preference of androgen-responsive lncRNAs (Figure 2(c)). Most of differentially expressed lncRNAs are transcribed from the intergenic regions (Figure 2(d)).

### 3.2. Validation of Androgen-Responsive lncRNAs in LNCaP Cells

**Figure 3:**
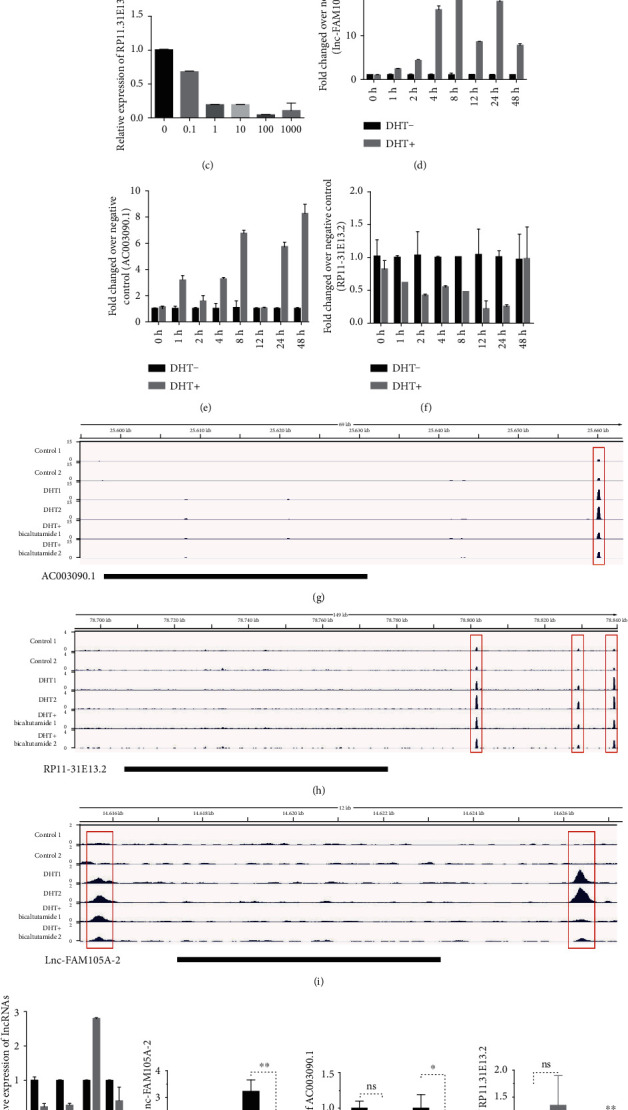
The validation of androgen-responsive lncRNAs in LNCaP cells. (a–c) RT-PCR analyses of lnc-FAM105A-2, AC003090.1, and RP11-31E13.2's expressions in LNCaP cells treated with DHT for 24 h in dose series of 0 nM, 0.1 nM, 1 nM, 10 nM, 100 nM, and 1000 nM. Values of expressions treated with equal volume of vehicle were used as control. (d–f) RT-PCR analyses of lnc-FAM105A-2, AC003090.1, and RP11-31E13.2's expressions in LNCaP cells treated with 10 nM DHT in time series of 0 h, 2 h, 8 h, 24 h, and 48 h. Values of expressions treated with equal volume of vehicle in the same time series were used as control. (g–i) Genome browser view of the AC003090.1, RP11-31E13.2, and lnc-FAM105A-2 genomic locus for AR ChIP-seq data tracks obtained from VCaP cells treated with either vehicle or dihydrotestosterone (DHT) alone or combinations including DHT+Bicalutamide. Significant AR binding observed in each data track are represented as peaks. (j) The expressions of lnc-FAM105A-2 and AC003090.1 and RP11-31E13.2 after AR knockdown compared with NC in LNCaP cells. (k–m) The expressions of lnc-FAM105A-2, AC003090.1, and RP11-31E13.2, after AR knockdown in LNCaP cells with or without DHT treatment. Results are presented as the mean ± sd of three independent experiments. Significance was defined as *P* < 0.05 (^∗^*P* < 0.05, ^∗∗^*P* < 0.01, and ^∗∗∗^*P* < 0.001).

Three lncRNAs (lnc-FAM105A-2, AC003090.1, and RP11-31E13.2) with significant expression changing were randomly selected for further qRT-PCR validation. To further validate array data, we also detected lnc-FAM105A-2 (Figure 3(a)), AC003090.1 (Figure 3(b)), and RP11-31E13.2 (Figure 3(c)) expressions in LNCaP cells afer 8 h treatment with different doses of DHT (0, 0.1, 1, 10, 100, and 1000 nM). We also detected the expression of these lncRNAs after DHT treatment in a time-dependent assay. lnc-FAM105A-2 expression was also significantly elevated (25-fold in 8 h and 20-fold in 24 h) upon DHT stimulation (Figure 3(d)). AC003090.1 expression was significantly upregulated (sixfold in 24 h and eightfold in 48 h) upon DHT stimulation (Figure 3(e)). However, we observed that RP11-31E13.2 was significantly downregulated (20 percent in 12 h and 24 h) after DHT treatment (Figure 3(f)). We observed the same expression tendency under DHT stimulation in a dose series. These results were consistent with microarray data.

To test the hypothesis that these lncRNAs were AR targets, we assessed the presence of AR peaks in AC003090.1, lnc-FAM105A-2, and RP11-31E13.2 genomic region using AR-ChIP-seq data generated in VCaP cells [16]. We found that AR peaks in these lncRNA loci significantly increased after DHT treatment and also observed that these peaks were suppressed upon treatment with AR antagonist bicalutamide (Figures 3(g)–3(i)). Next, we detected AC003090.1, lnc-FAM105A-2, and RP11-31E13.2 after AR knockdown (Figure 3(j)). Notably, we found that lnc-FAM105A-2 and AC003090.1 were significantly downregulated and the expression of RP11-31E13.2 was induced after AR silencing which depends on DHT treatment (Figures 3(k)–3(m)).

### 3.3. Construction of Upstream Transcriptional Regulatory Network of Androgen-Responsive lncRNAs

**Figure 4:**
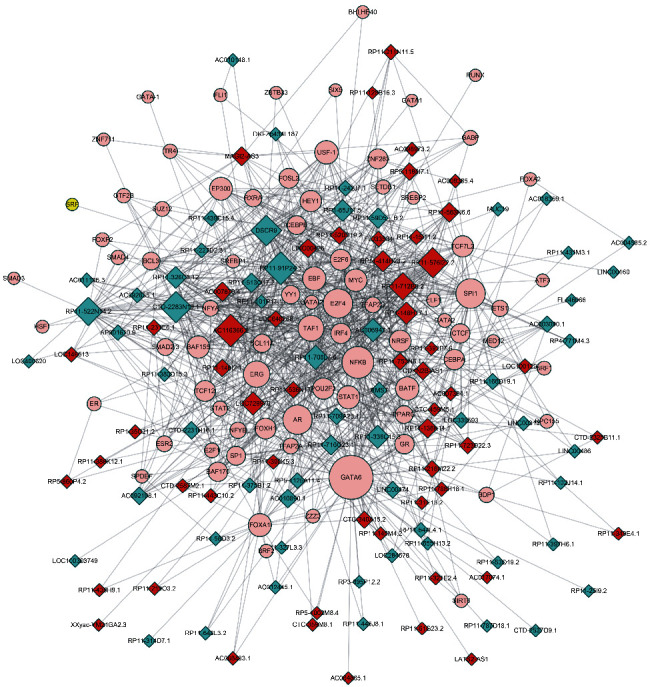
The clusters of upstream transcriptional regulatory network of androgen-responsive lncRNAs for identifying unknown transcriptional factors. Diagram analyses ChIP-seq data from ChIPbase. Construction of upstream transcriptional network that regulate androgen-responsive lncRNA expression. Circle: core TFs; red diamond: androgen-reduced lncRNAs; blue diamond: androgen-induced lncRNAs.

In the present study, we found only 30% androgen-responsive lncRNAs had AREs, suggesting that other transcriptional factors may also participate in regulating lncRNAs expression in response to DHT. To construct upstream transcriptional regulatory network of androgen-responsive lncRNAs, we used ChIP-seq data from ChIP base. Thirty TFs including AR, GATA6, and NFKB were identified. Cytoscape 3.0 was used to draw the transcriptional regulatory network. Interestingly, some TFs such as ERG, EP300, and FOXA1 had been reported as AR targets or cofactors (Figure 4(a)).

### 3.4. Construction of Androgen-Responsive ceRNA Networks in PCa

One of the most important functions of lncRNAs was competitively binding to miRNA to affect protein-coding genes translation and posttranscriptional regulation. Thus, we constructed an androgen-responsive ceRNA network in PCa, combined with our previous identified androgen-responsive mRNAs and miRNAs datasets. We predicted the interactions between differentially expressed lncRNAs and their target miRNAs theoretically by using miRcode. Finally, TargetScan and StarBase databases were both used to identify miRNAs which suppress mRNAs. The networks were drawn using Cytoscape 3.0 (Figures 1(a)–1(d)).

### 3.5. The Expression Patterns of Androgen-Responsive lncRNAs in Human Tissues

**Figure 5:**
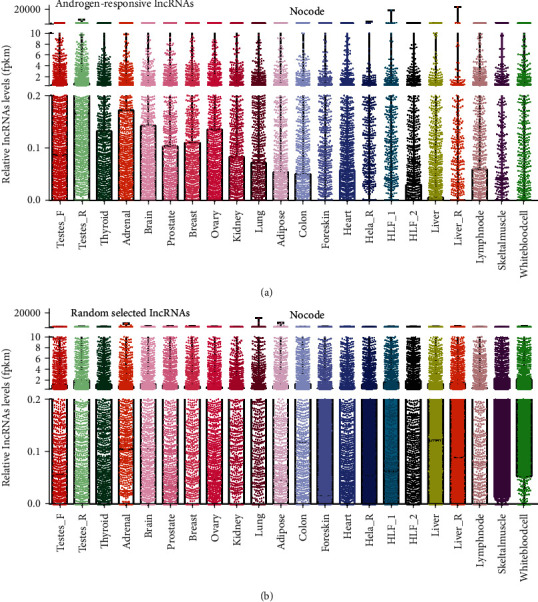
The expression patterns of androgen-responsive lncRNAs in human tissues. The relative expression levels plot in testes, thyroid, adrenal, prostate, breast, ovary, kidney, and lung were showed for (a) androgen-responsive lncRNAs and (b) randomly selected lncRNAs. We observed that androgen-responsive lncRNAs were overexpressed in in hormone-related tissues such as testes, thyroid, adrenal, prostate, breast, and ovary.

To better understand the tissue-specific expression characteristics of androgen-responsive lncRNAs in different human tissues, we analyzed RNA sequencing profiles (RNA-seq) from 22 different human tissues in NOCODE database. Interestingly, we found androgen-responsive lncRNAs were significantly overexpressed in testes, thyroid, adrenal, prostate, breast, ovary, kidney, and lung (Figure 5(a)). We also compared the expression levels of the same number of lncRNAs which were randomly selected and found the expression of these randomly selected lncRNAs in 22 different human tissues were the same (Figure 5(b)). These results revealed the potential important roles of androgen-responsive lncRNAs in hormone-related tumors.

### 3.6. The Expression of Androgen-Responsive lncRNAs Is Dysregulated in Hormone-Related Cancers

**Figure 6:**
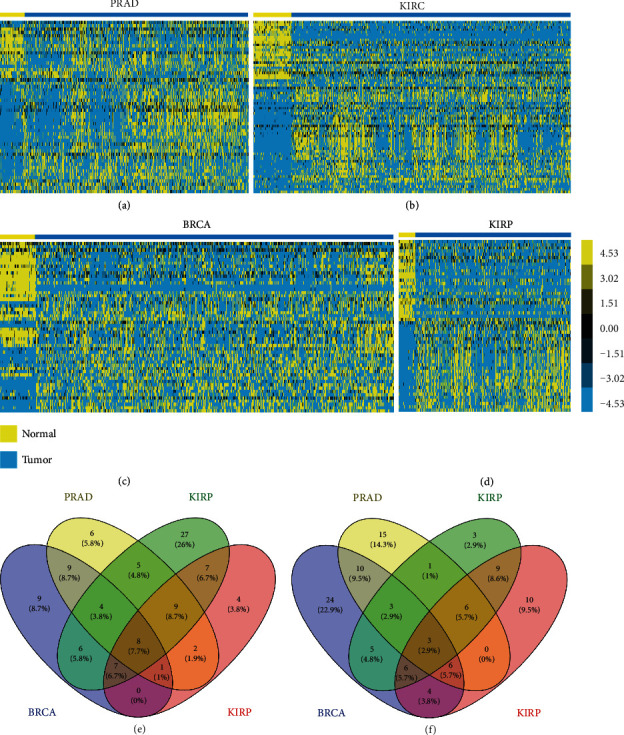
The dysregulated androgen-responsive lncRNAs in hormone-related cancers. The heat map of RNA sequencing profiles (RNA-seq) in cancer hormone-related tumors including (a) prostate cancer (PRAD), (b) kidney renal clear cell cancer (KIRC), (c) breast cancer (BRCA), and (d) kidney renal papillary cell cancer (KIRP). The overlap of four hormone-related tumors for (e) upregulated and (f) down-regulated lncRNAs.

To characterize tumor-associated dysregulation of androgen-responsive lncRNA expression, we analyzed RNA sequencing profiles (RNA-seq) from 2037 tumors across 6 hormone-related tumors, including prostate cancer (PRAD), breast cancer (BRCA), kidney renal clear cell cancer (KIRC), kidney renal papillary cell cancer (KIRP), ovarian serious cystadenocarcinoma (OV), and testicular germ cell tumors (TGCT) in TCGA. The information of clinical samples is listed in supplementary table 2. However, we did not find normal samples in OV and TGCT datasets. Compared to their normal samples, we found that more than 45% lncRNAs were significantly up- or downregulated in PRAD (48%), BRCA (58%), KIRC (60%), and KIRP (45%) samples, indicating the important roles of androgen-responsive lncRNAs in these cancers (Figures 6(a)–6(d)). By comparing the dysregulated lncRNAs in different cancer types, we found that ~10% of these altered lncRNAs were cancer-type specific, and the rest were shared by at least two cancer types. We identified only 8 lncRNAs was upregulated and 3 lncRNAs was downregulated in all four cancer types (Figures 6(e) and 6(f)). The lncRNAs whose dysregulated expression was shared or unique among different cancer types are listed in supplementary table 3.

### 3.7. Biological Functions of Androgen-Responsive lncRNAs in Hormone-Related Cancers

**Figure 7:**
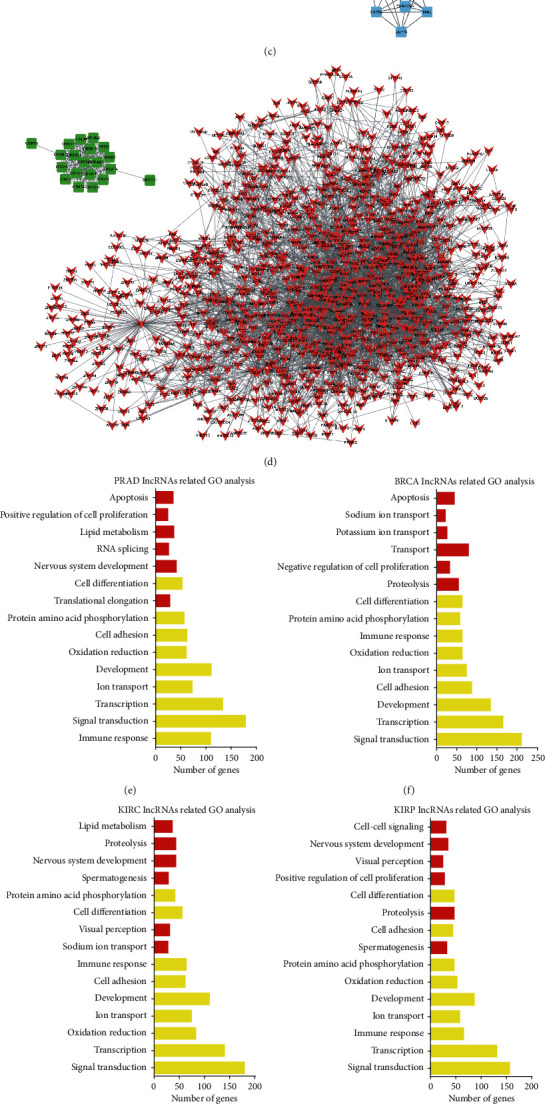
The biological functions of androgen-responsive lncRNAs in hormone-related cancers. The clusters of androgen-responsive lncRNAs mediated PPI networks in hormone-related cancers including (a) prostate cancer (PRAD), (b) breast cancer (BRCA), (c) kidney renal clear cell cancer (KIRC), and (d) kidney renal papillary cell cancer (KIRP). The GO analysis of lncRNAs in (e) PRAD, (f) BRCA, (g) KIRC, and (h) KIRP. The KEGG pathway analysis of lncRNAs in (i) PRAD, (j) BRCA, (k) KIRC, and (l) KIRP using the set of coexpressed mRNAs.

To predict the functions of the androgen-responsive lncRNAs in hormone-related cancers, we adopted methods as described by Guttman et al. and Shen et al. We first constructed coexpression analysis to identify the correlation between differentially expressed mRNAs and lncRNAs. Next, we mapped the androgen-responsive lncRNAs coexpressed genes to STRING with experimentally validated interactions score > 0.4. Then, PPI networks were constructed using the Cytoscape software. Interestingly, we found lncRNAs mediated PPI networks in different kinds of cancers were not the same; however, a PPI network formed by olfactory receptor family existed in PRAD (Figure 7(a)), BRCA (Figure 7(b)), KIRC (Figure 7(c)), and KIRP (Figure 7(d)). The olfactory receptor proteins are members of GPCR family and shared a 7-transmembrane domain structure with many neurotransmitter and hormone receptors.

Next, we performed GO and KEGG pathway analyses for each given lncRNA using the set of coexpressed mRNAs (Figures 7(e)-7(l)). In this study, the top 50 related mRNAs of each lncRNAs were classified according to GO terms. The top 15 generally changed biological processes were listed. GO analysis revealed that 9 biological processes (including signal transduction, transcription, development, cell adhesion, ion transport, oxidation reduction, immune response, protein amino acid phosphorylation, and cell differentiation) were widely regulated by androgen-responsive lncRNAs in PRAD (Figure 7(e)), BRCA (Figure 7(f)), KIRC (Figure 7(g)), and KIRP (Figure 7(h)). We also observed some androgen-responsive lncRNAs-regulated biological processes were cancer specific, for example, RNA splicing and translational elongation in PRAD, cell-cell signaling, visual perception and spermatogenesis in KIRP and KIRC, and negative regulation of cell proliferation, transport, and potassium ion transport in BRAD.

KEGG analysis showed that androgen-responsive lncRNAs in PRAD were enriched in the calcium signaling pathway, T cell receptor signaling pathway, MAPK signaling pathway, Jak-STAT signaling pathway (Figure 7(i)). Androgen-responsive lncRNAs in BRCA were enriched in the MAPK signaling pathway, PPAR signaling pathway, T cell receptor signaling pathway (Figure 7(j)), GnRH signaling pathway. Androgen-responsive lncRNAs in KIRC were enriched in the T cell receptor signaling pathway, Pentose phosphate pathway, MAPK signaling pathway, PPAR signaling pathway (Figure 7(k)). GO analysis revealed that androgen-responsive lncRNAs in KIRP were enriched in the MAPK signaling pathway and calcium signaling pathway (Figure 7(l)).

### 3.8. Prognostic Significance of Androgen-Responsive lncRNAs in Hormone-Related Cancers

**Figure 8:**
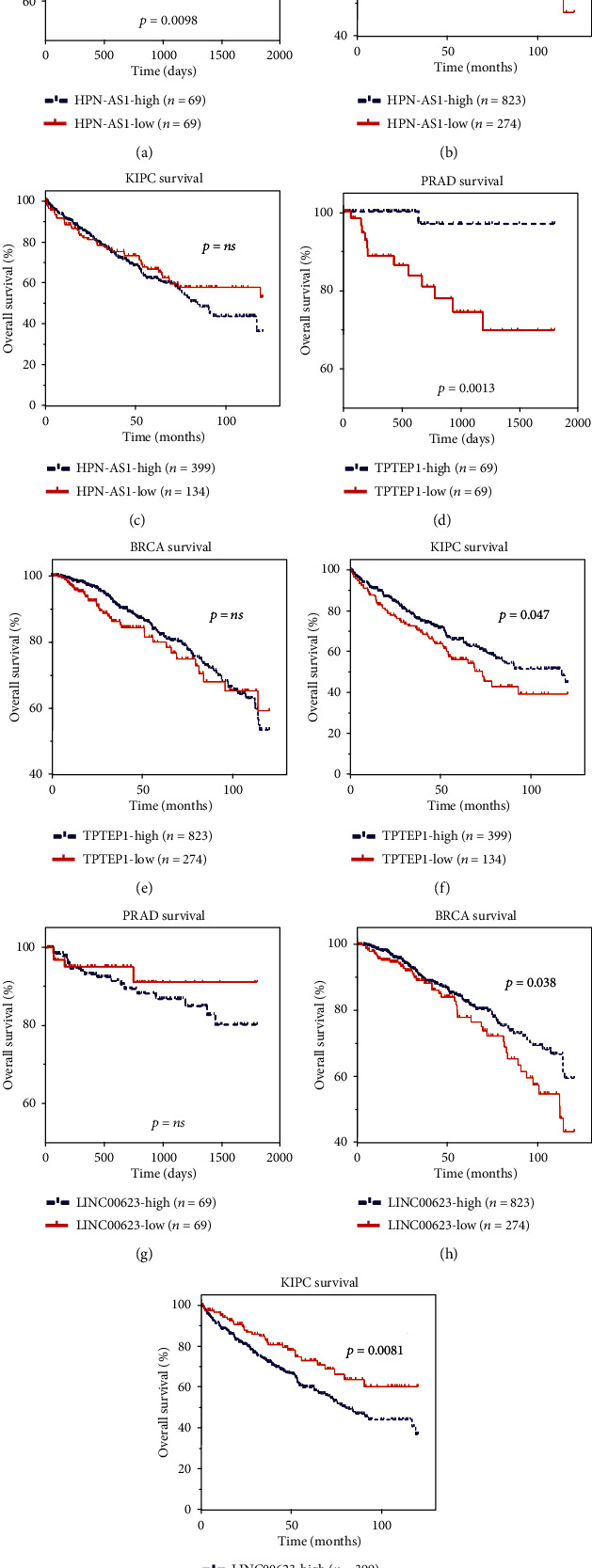
Androgen-responsive lncRNAs' pathologic features in hormone-related cancers. (a–c) Kaplan-Meier curve analysis for the correlation between HPN-AS1 expression and disease-free survival time in patients with PRAD, BRAC, and KIRC. (d–f) Kaplan-Meier curve analysis for the correlation between TPTEP1 expression and disease-free survival time in patients with PRAD, BRAC, and KIRC. (g–i) Kaplan-Meier curve analysis for the correlation between LINC00623 expression and disease-free survival time in patients with PRAD, BRAC, and KIRC. Significance was defined as *P* < 0.05 (^∗^*P* < 0.05, ^∗∗^*P* < 0.01, and ^∗∗∗^*P* < 0.001).

To evaluate possible prognostic value of androgen-responsive lncRNAs in hormone-related cancers, we analyzed TCGA database and found that these lncRNAs showed significant association with cancer progression. Of note, we found three lncRNAs (HPN-AS1, TPTEP1, and LINC00623) could serve as biomarkers for PRAD (Figures 8(a), 8(d), and 8(g)), BRCA (Figures 8(b), 8(e), and 8(h)), and KIRC (Figures 8(c), 8(f), and 8(i)). Kaplan-Meier analysis showed that the BCR-free survival rates were lower in HPN-AS1-high, TPTEP1-low, and LINC00623-high groups in PRAD and KIRC patients. Interestingly, we observed the high levels of TPTEP1 and LINC00623 were correlated with a longer biochemical recurrence-free survival times in BRAC.

## 4. Discussion

AR signaling plays important roles in regulating tumorigenesis and metastasis in several cancers including, kidney, lung, breast, and testis cancer [17]. LncRNAs have been reported as important regulators of cell growth, proliferation, and differentiation in many types of cancer, such as prostate, bladder, and kidney cancer [18]. Our group and others have focused on exploring the molecular functions of androgen-responsive lncRNAs in PCa. However, there is still a lack of extensively identification of androgen response lncRNAs. In this study, we focused on androgen-responsive lncRNAs to determine their roles in hormone-related cancers. To identify androgen-responsive lncRNAs, we performed high-throughput microarray assay in PCa cell line LNCaP. A total of 285 lncRNAs were upregulated and 184 lncRNAs were downregulated after DHT treatment. Three lncRNAs (AC003090.1, lnc-FAM105A-2, and RP11-31E13.2) with significant expression change were randomly selected for further qRT-PCR validation. Our qRT-PCR results were consistent with microarray data. Importantly, ChIP-seq data showed AR peaks in these lncRNA loci significantly increased after DHT treatment and these peaks were suppressed upon treatment with AR antagonist bicalutamide, which suggest these lncRNAs were direct targets of AR.

The functional importance of lncRNA has been paid more and more attention by researchers. Recently, a few studies had indicated some transcriptional factors, including EZH2 [19], AR, MYC [20], and ER*α* [21], could regulate lncRNAs' expression. However, the transcriptional regulation of the majority of lncRNAs remained unknown. Our groups had identified a series of androgen-responsive lncRNAs [12]. Of note, in our previous study, agilent human lncRNA array was performed to simultaneously observe lncRNA expressions in androgen-dependent LNCaP cells under DHT stimulation in time points of 0 h and 2 h, respectively. The “0 h” worked as the control representing cellular status before DHT stimulation. Supervised analysis of the microarray data showed 3767 deregulated lncRNA transcripts (1991 transcripts upregulated and 1776 transcripts down-regulated) produced from 2980 genes, with an average expression level over 2-fold change in 2 h compared to 0 h (GSE72866). In this study, high-throughput microarray assay (SBC-ceRNA (4^∗^180 K)). was performed to identify androgen-responsive lncRNAs in LNCaP cells after 8 h treatment of DHT. Several differences between the both studies were observed. First, the platform used in both studies was different. Second, our previous studies mainly aimed to identify early response lncRNAs. This mainly aimed to identify late response lncRNAs. We also found that more than 50 percent androgen-responsive lncRNAs did not contain AREs, suggesting that other TFs may also be involved in regulating lncRNA expression. Interestingly, Crea et al. also found that androgen-regulated PCAT18 was not a direct target of AR [22]. To comprehensively reveal upstream transcriptional regulatory network of androgen-responsive lncRNAs, we analyzed ChIP-seq data from ChIPbase.

More than 30 TFs were identified in our transcriptional network. Among them, AR, GATA6, and NFKB showed the most widely regulation of androgen-responsive lncRNAs. Interestingly, we also identified that many hormone receptors (such as ER and GR) and AR-cofactors (including FOXA1, EP300, and GR) participated in regulating these lncRNAs. By analyzing our previous reports, we found that 85 percent of these transcriptional factors were also regulated by AR.

To understand the tissue-specific expression characteristics of androgen-responsive lncRNAs, we analyzed their expressions in 22 different human tissues and found androgen-responsive lncRNAs were significantly overexpressed in testes, thyroid, adrenal, prostate, breast, ovary, kidney, and lung. Hormone-related cancers, such as prostate cancer and breast cancer, were the most commonly diagnosed cancers in the worldwide. Accumulating evidence demonstrated that AR also played key roles in regulating hormone-related cancer progression. In the previous reports, a series of lncRNAs (such as CTBP1-AS in PCa [13], lncARSR in renal cancer [23], and NKILA in breast cancer [24] were reported to play key roles in regulating cancer progression. However, there were no reports that revealed androgen-responsive lncRNA roles in these cancers.

To characterize tumor-associated dysregulation of androgen-responsive lncRNAs' expression, we analyzed RNA sequencing profiles (RNA-seq) from 2037 tumors across 6 cancer hormone-related tumors (PRAD, BRCA, KIRC, KIRP, OV, and TGCT) in TCGA and found that more than 45% lncRNAs were significantly up- or downregulated in PRAD (48%), BRCA (58%), KIRC (60%), and KIRP (45%) samples, indicating the important roles of androgen-responsive lncRNAs in these cancers. To predict the functions of the androgen-responsive lncRNAs in hormone-related cancers, we performed GO and KEGG pathway analyses using the set of coexpressed mRNAs. Our results showed that androgen-responsive lncRNA played different roles in hormone-related cancers. Eight lncRNAs were upregulated, and 3 lncRNAs were downregulated in all four cancer types. To evaluated prognostic values of differentially expressed androgen-responsive lncRNAs in hormone-related cancers, we then analyzed TCGA database and found that three lncRNAs (HPN-AS1, TPTEP1, and LINC00623) could serve as biomarkers for PRAD, BRCA ,and KIRC due to their expression levels, which were significantly correlated to overall survival.

In conclusion, our study represents the comprehensive analysis of androgen-responsive lncRNAs in prostate cancer. To reveal the upstream regulating factors, we constructed transcriptional network of lncRNAs for the first time. We also analyzed the expression patterns of lncRNAs in 22 different kinds of human tissues. Of note, we found that androgen-responsive lncRNAs were dysregulated in hormone-related cancers such as PRAD, BRCA and KIRC. GO and KEGG pathway analyses showed that androgen-responsive lncRNAs played different roles in different hormone-related cancers. We also highlight the prognostic value of HPN-AS1, TPTEP1, and LINC00623 in cancer outcomes. Further studies, however, will be needed to develop a deeper understanding of the mechanistic roles of androgen-responsive lncRNAs in the development and progression of cancer.

## Data Availability

The data used to support the findings of this study are available from the corresponding authors upon request.

## Supplementary Materials

Supplementary 1 the raw microarray data were listed in supplementary table 1.

Supplementary 2 the information of clinical samples was listed in supplementary table 2.

Supplementary 3 the lncRNAs whose dysregulated expression was shared or unique among different cancer types are listed in supplementary table 3.
